# DNA methylation profiling in Trisomy 21 females with and without breast cancer

**DOI:** 10.3389/fonc.2023.1203483

**Published:** 2023-07-19

**Authors:** Yosra Bejaoui, Sara Alresheq, Sophie Durand, Marie Vilaire-Meunier, Louise Maillebouis, Ayman Al Haj Zen, André Mégarbané, Nady El Hajj

**Affiliations:** ^1^ College of Health and Life Sciences, Hamad Bin Khalifa University, Qatar Foundation, Doha, Qatar; ^2^ Institut Jérôme Lejeune, Paris, France; ^3^ Department of Human Genetics, Gilbert and Rose-Marie Chagoury School of Medicine, Lebanese American University, Byblos, Lebanon; ^4^ College of Science and Engineering, Hamad Bin Khalifa University, Qatar Foundation, Doha, Qatar

**Keywords:** Down syndrome, breast cancer, DNA methylation, epigenetics, Trisomy 21

## Abstract

**Background:**

Down Syndrome (DS) is the most common chromosome anomaly in humans and occurs due to an extra copy of chromosome 21. The malignancy profile in DS is unique, since DS patients have a low risk of developing solid tumors such as breast cancer however they are at higher risk of developing acute myeloid leukemia and acute lymphoblastic leukemia.

**Methods:**

In this study, we investigated DNA methylation signatures and epigenetic aging in DS individuals with and without breast cancer. We analyzed DNA methylation patterns in Trisomy 21 (T21) individuals without breast cancer (T21-BCF) and DS individuals with breast cancer (T21-BC), using the Infinium Methylation EPIC BeadChip array.

**Results:**

Our results revealed several differentially methylated sites and regions in the T21-BC patients that were associated with changes in gene expression. The differentially methylated CpG sites were enriched for processes related to serine-type peptidase activity, epithelial cell development, GTPase activity, bicellular tight junction, Ras protein signal transduction, etc. On the other hand, the epigenetic age acceleration analysis showed no difference between T21-BC and T21-BCF patients.

**Conclusions:**

This is the first study to investigate DNA methylation changes in Down syndrome women with and without breast cancer and it could help shed light on factors that protect against breast cancer in DS.

## Introduction

Down syndrome (DS) is a genetic disorder caused by an additional copy of all or part of chromosome 21 resulting in 47 chromosomes instead of the typical 46 chromosomes. The etiology of DS was identified following the discovery of karyotyping techniques when the French geneticist Jérôme Lejeune reported that an extra chromosome 21 results in the phenotypic features and intellectual disability associated with DS (1). DS is considered the most common chromosomal condition in humans occurring in 1 out of every 700 newborn babies (2). DS has three different forms including Trisomy 21 (nondisjunction), mosaicism, and translocation. Nondisjunction of chromosome 21, also called standard trisomy 21, is the most common DS type and accounts for ~ 95% of all cases. The cause of this chromosomal non-disjunction occurs mainly during maternal meiotic division (~88% of the cases). Whereas, ~ 5-10% of the cases are caused by non-disjunction during spermatogenesis and a small percentage of cases are due to mitotic error or occur during the first mitotic divisions of the embryo (3–5).

Trisomy 21 is associated with more than 100 features including intellectual disability, distinctive facial features, early aging, neurodegeneration, and muscle hypotonia during childhood (6). Intellectual disability is the most common feature in DS patients, where it usually ranges from mild to moderate. Besides, DS patients have a high incidence of congenital heart disease, early onset Alzheimer’s disease, gastrointestinal and skeletal malformations, and a diversity of neurobehavioral abnormalities (7–9). Even though DS patients are predisposed to developing acute lymphoblastic and myeloblastic leukemia during childhood, solid tumors seem to be extremely rare in both children and adults (10–16). Several epidemiological studies suggested that the risk of developing solid tumors in DS patients is at least 12 times lower than that of the general population (16, 17). For example, breast cancer (BC) is almost non-existent in DS females, despite genetic instability, deficiencies in DNA repair, increased oxidative stress, sedentary lifestyle, higher obesity rates, and increased DNA damage. Environmental factors including decreased exposure to estrogens and low alcohol consumption are not sufficient by themselves to explain the low rate of BC in DS females (17–19). Therefore, it would be important to study possible molecular mechanisms that protect against the development of breast cancer in Down syndrome.

Epigenetic dysregulation in response to an additional copy of chromosome 21 has been reported to affect the entire genome and not only genes located on chromosome 21 (20–24). Those changes arise during development and systemically affect multiple tissues (21, 25). Epigenetic clocks based on DNA methylation measurements have been used to estimate a person’s biological age and epigenetic aging acceleration. Epigenetic age acceleration has been reported to be associated with cancer risk, prognosis, and survival (26). Furthermore, patients with Down syndrome were reported to have drastic epigenetic age acceleration that was even higher than in certain progeroid syndromes (27, 28). Taking into account the occurrence of epigenetic alterations in most cancers and that they act as drivers to cancer progression, it would be important to study whether DNA methylation alterations affecting certain genes/pathways confers protection against breast cancer in DS. Therefore, we performed a genome-wide DNA methylation analysis in DS females with and without BC to determine epigenetically dysregulated regions linked to the lower BC frequency in DS. In addition, we compared epigenetic age acceleration in DS females with and without BC.

## Materials and methods

### Samples and data collection

A total of 5532 files were screened at the Jérôme Lejeune Institute (CRB BioJeL, Paris, France) to identify two DS females with homogeneous Trisomy 21 (T21) diagnosed with breast cancer (no mosaicism or translocation cases were included). Sequencing analysis revealed no pathogenic or likely pathogenic variants in genes associated with an increased risk of breast cancer in the selected samples. A total of 10 age matched DS females with homogeneous T21 and without breast cancer (or any mammary lesion) were selected as controls (**Supplementary Table 1**). All the recruited DS women were > 34 years old, without any chronic medications or social problems. No breast cancer was recorded in the families of the DS women in this study. Whole blood samples were collected from all the patients and human peripheral blood mononuclear cells (PBMCs) were isolated. DNA was extracted from both whole blood and PBMCs. Written informed consent was obtained from the parents or guardians for all participants included in the research study.

### DNA methylation quantification using EPIC arrays

DNA methylation profiling was performed for two T21 females with breast cancer (referred to as T21-BC) and for 10 T21 females without breast cancer (T21-BCF) (n=10) using the Illumina Infinium Epic array. DNA samples were processed on Illumina Infinium Epic array according to the manufacturer’s protocol. Briefly, 500 ng DNA for each sample was bisulfite converted using the EZ DNA Methylation Kit (Zymo Research, Irvine, CA, USA). Afterwards, bisulfite converted DNA was whole-genome amplified, enzymatically fragmented, and hybridized to Infinium Methylation EPIC BeadChips. Array scanning was performed via the Illumina iScan. To avoid batch effects, all samples were processed simultaneously and measured samples were randomly hybridized on the arrays. Idat files were exported and analyzed with the R software package (version 3.2.2) and the BioConductor platform (version 3.2).

### Differential DNA methylation analysis

The RnBeads package was used for differential methylation analysis (29). First, the data quality was assessed and probes mapping to multiple regions in the genome (Cross-reactive) or overlapping SNPs were removed. Furthermore, probes with unreliable measurements were removed via greedycut prior to further analysis. Next, additional filtering of polymorphic probes in the European, admixed American, South and East Asian, and African was applied using “filtering.blacklist” option (30). Data was normalized using Dasen and probes located on the X chromosome were retained because only females were analyzed. A total of 534862 (whole blood) and 534049 (PBMC) probes were finally retained for differential DNA methylation analysis. Inference for blood cell composition was performed using the Houseman method (31). Next, a limma based approach was used to correct for cell type composition, age, and surrogate variables. Differential methylation analysis was performed at the single CpG site level and at the level of promoters, CpG islands, and tiling windows (5Kb). Combined p-values were calculated and adjusted for multiple testing using false discovery rate (FDR) correction. Gene Ontology (GO) enrichment analysis was performed via the methylglm function from the methylGSA package (32).

### Calculating DNA methylation age and age acceleration

Epigenetic age acceleration was measured using several epigenetic clocks that utilize different CpG sites to estimate DNA methylation (DNAm) age using the DNAm age calculator (https://dnamage.genetics.ucla.edu/) with the normalization option selected.

### DNA methylation data from breast cancer patients with normal karyotype

DNA methylation profiles of women with normal karyotype diagnosed with breast cancer (n=43) were downloaded from NCBI’s Gene Expression Omnibus (GEO Series accession: GSE104942). The Raw (IDAT) files were processed as previously described in the “Differential DNA methylation analysis” section. In total, the studied dataset was comprised of blood DNA methylation data measured via the Illumina EPIC arrays on 43 Breast cancer patients and 12 controls.

## Results

### Differentially methylated sites in Down’s syndrome females with breast cancer

**Figure 1:**
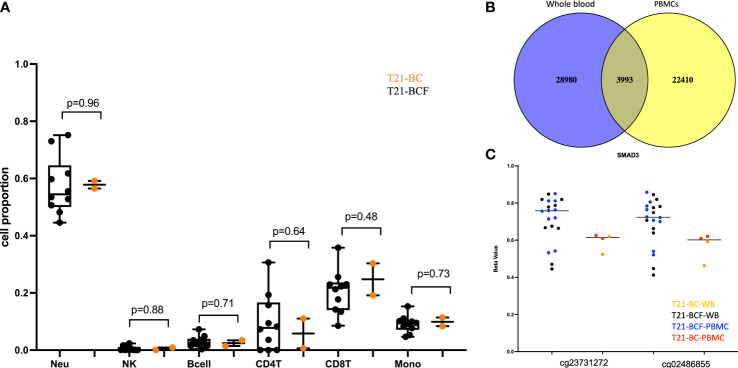
**(A)** Comparison of deconvoluted cell proportions measured via the Houseman method in whole blood of Trisomy 21 (T21) individuals with breast cancer (T21-BC) vs T21 without breast cancer (T21-BCF); **(B)** Ven-diagram of significant sites with unadjusted p-value <0.05 when comparing whole blood and PBMCs of T21-BC vs T21BCF; **(C)** differentially methylated probes s in *SMAD3* with more than 9% methylation differences in whole blood and PBMC and present in the list of differentially expressed genes in T21-BC performed on the same samples. T21-BC-WB: Trisomy 21 (T21) individuals with breast cancer whole blood analysis. T21-BCF-WB: Trisomy 21 (T21) individuals without breast cancer whole blood DNA methylation analysis. T21-BCF-PBMC and T21-BC-PBMC indicate the DNA methylation analysis in peripheral blood mononuclear cells.

To identify epigenetically altered regions associated with BC in DS, we measured DNA methylation levels in T21 breast cancer patients (T21-BC, n=2) vs T21 breast cancer-free patients (T21-BCF, n=10) using the Illumina EPIC arrays. DNA methylation was profiled in DNA isolated from both whole blood and from PBMCs. The number of T21-BC samples was limited because only two T21 females with breast cancer were identified after screening 5532 files at Jérôme Lejeune Institute. For this reason, we decided to measure DNA methylation in duplicates across both whole blood and PBMC samples. First, we compared the deconvoluted blood cell proportions in T21-BC vs. T21-BCF as estimated by the Houseman method, which revealed no change in immune blood cell proportions (**Figure 1A**).

**Figure 2:**
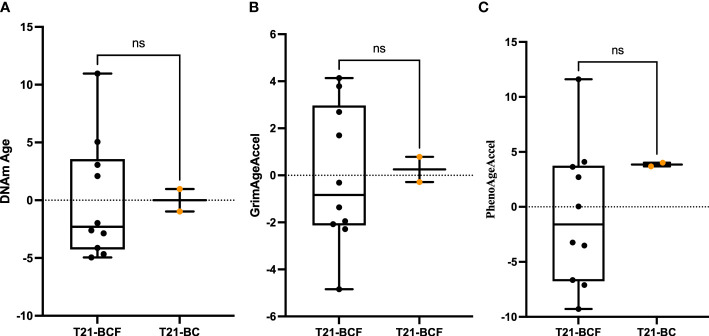
Epigenetic age acceleration in whole blood DNA of T21-BC vs T21-BCF using the **(A)** Horvath, **(B)** PhenoAge and, **(C)** GrimAge clocks. DNA methylation (DNAm) age acceleration, which represents the residual of regressing epigenetic age on chronological age is shown on the y-axes. ns, not significant.

Next, differential DNA methylation analysis was performed to compare T21-BC vs. T21-BCF. The differential methylation was assessed primarily at the CpG sites level in addition to the region level including promoter, CpG Island, and tiling regions using a 5Kb sliding window. We did not observe any significance at the CpG site or the region level after FDR adjustment when adjusting for age, gender, cell type composition, and surrogate variables. This could be related to the low sample number in the T21-BC group. For this reason, we looked at common significant sites/regions with unadjusted p-value <0.05 between T21-BC and T21-BCF. In total, 32973 and 26403 CpG sites were significant before FDR adjustment in WB and PBMCs, respectively. Out of which, 3993 CpG sites were common between the whole blood and PBMC samples (**Figure 1B**). Out of those, 3087 had a similar direction of DNA methylation change when comparing T21-BC and T21-BCF in both WB and PBMC samples. When we filtered for ≥ 3% methylation in both tissues, a total of 1601 CpG sites were retained. Next, we applied the methylGSA package to test for gene ontology (GO) and KEGG pathway enrichment in those CpG sites, after adjusting for probe bias distribution across genes in the EPIC arrays. The GO enrichment analysis revealed several significant terms including serine-type peptidase activity, exopeptidase activity, serine hydrolase activity, epithelial cell development, *etc* (**Supplementary Table 2**). On the other hand, the KEGG analysis did not reveal any pathway enrichment for the 1601 CpG sites. We additionally investigated epigenetic age acceleration in T21-BC vs T21-BCF using the Horvath clock, GrimAge and PhenoAge, which revealed no DNA methylation age acceleration difference T21-BC in whole blood (**Figure 2**) and PBMC samples.

### Differentially methylated regions associated with breast cancer in Down’s syndrome

Next, we looked at the promoter region where we could identify 832 significant promoters (unadjusted p-value <0.05) in whole blood and 744 significant promoters in PBMCs. A total of 78 promoters were significant in both analyses when comparing whole blood and PBMCs from T21-BC vs T21-BCF with the same direction of methylation change. Out of which, 22 promoters had > 2 CpG sites and ≥ 3% methylation in both whole blood and PBMC samples (**Supplementary Table 3**). For the CpG Island analysis, we could observe 131 common significant CGIs with the same direction of methylation change, including 43 with > 2 CpG sites and ≥ 3% methylation (**Supplementary Table 4**). For the tiling analysis, we could identify 677 regions (5Kb) differentially methylated in a similar direction in both datasets, however only 79 remained after filtering using the previously defined criteria (≥ 2 CpG sites, ≥ 3% methylation). Next, we tested whether the identified DMPs/DMRs are similarly epigenetically dysregulated in blood DNA of breast cancer patients. The studied dataset (GSE104942) contained blood DNA methylation data of 43 Breast cancer patients and 12 healthy controls. This analysis revealed no common significant DMRs between the T21-BC list and the differentially methylated genes in breast cancer patients. Two DMPs (cg05997779 and cg26845300) were similarly epigenetically altered in both datasets, however, they exhibited different direction of DNA methylation change.

### Transcriptional changes in epigenetically dysregulated genes associated with breast cancer in Down’s syndrome

**Figure 3:**
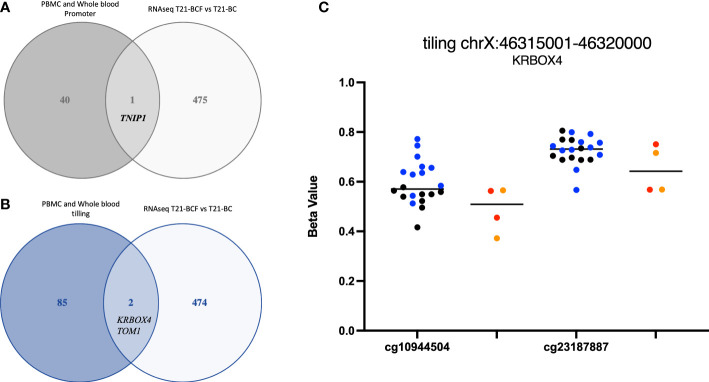
Ven-diagram displaying **(A)** differentially methylated promoters and **(B)** differentially methylated regions following a tiling analysis of differentially expressed genes in Trisomy 21 (T21) individuals with breast cancer (T21-BC) vs T21 without breast cancer (T21-BCF). **(C)** *KRBOX4* DNA methylation distribution in T21 BC vs T21 BCF. Genomic coordinates based on genome assembly GRCh37 (hg19).

Finally, we compared the differentially methylated sites/regions to the list of 183 differentially expressed genes in T21-BC identified following RNA-seq on the same samples (33). Here, we could observe 37 differentially methylated probes (DMPs) associated with differentially expressed genes and same direction of methylation change in both DNA methylation datasets. When we filtered for ≥ 3% methylation difference, we could only detect 12 CpG sites that fit this criteria including two close DMPs in *SMAD3* with more than 9% methylation differences in all comparisons (**Figure 1C**). In addition, there was a single CpG site located on chromosome 21 in the *BACH1* gene. Next, we checked the promoter and tiling differentially methylated regions (DMR), which revealed one DMR in the promoter analysis and two in the tiling region analysis. The common promoter was located in the gene TNFAIP3 Interacting Protein 1 (*TNIP1*) (**Figure 3A**), whereas the 5Kb tiling regions were located in the KRAB box domain containing 4 (*KRBOX4*) and Target Of Myb1 Membrane Trafficking Protein (*TOM1*) gene (**Figures 3B, C**). None of the CpG Island associated genes were differentially expressed in T21-BC.

## Discussion

To understand whether epigenetic dysregulation might explain the lower frequency of breast cancer in DS, we profiled DNA methylation in 12 women with T21 including two with breast cancer and 10 without breast cancer. DNA methylation was measured in both whole blood DNA and PBMCs as replicates due to the small number of breast cancer patients with T21.

The differential methylation analysis at the single CpG site level revealed 1601 DMPs with the same direction in methylation change. The gene ontology analysis revealed enrichment for serine-type peptidase activity, exopeptidase activity, serine hydrolase activity, epithelial cell development, endothelium development, transcription coactivator activity, GTPase activity, GTP binding, bicellular tight junction, and Ras protein signal transduction. Cell surface anchored serine proteases are deregulated in cancer cells and contribute to tumour invasion and metastasis (34). Evidently, Ras protein signal transduction is extremely important in cancer where mutations in the RAS genes were the first mutations reported in human cancers (35–37). The expression and activity small GTPases subfamily of “Ras-homology” (Rho) GTPase are known to be linked with breast tumour progression, angiogenesis, and metastasis (38). Similarly, bicellular tight junctions play a role in the epithelial-mesenchymal transition, which is essential in cancer progression (39). Excessive angiogenesis is a crucial component of tumour growth, invasiveness, and metastasis (40). The individuals with DS showed an elevated expression of *DSCR1* on the extra copy of chromosome 21, which is known to inhibit the growth of new blood vessels “angiogenesis” by suppressing vascular endothelial growth factor (VEGF)-mediated angiogenic signalling (41). In the current study, GO was enriched for endothelium development in which many genes overlap with angiogenesis. In addition, the crosstalk between several Rho GTPases and VEGF signalling is essential to control the process of angiogenesis (42–45). The epigenetic dysregulation in DS due to dosage imbalance of an additional chromosome 21 has been reported to occur extensively throughout the genome and is not restricted to genes located on chromosome 21 (21). This might lead to DNA methylation alterations in genes associated with the previously mentioned GO terms. This epigenetic dysregulation might confer protection to DS patients from breast cancer, which might help explain its reduced risk.

Furthermore, the comparison of the differentially methylated sites to differentially expressed genes following RNA-seq on the same set of samples identified 12 DMPs including two CpG sites in *SMAD3* with > 9% methylation difference. *Smad3* is a major transcription factor mediating transforming growth factor-β (TGF-β) signaling (46). The TGF‐β-Smad3 signaling has important roles in differentiation, apoptosis, and epithelial‐mesenchymal transition (EMT) (46, 47). SMAD-dependent signaling mediated by TGF-β has two opposing roles in cancer, where it first acts as a tumor suppressor in the initial phase, however in more advanced stages it is involved in inducing invasion and metastasis (48, 49). The regulation of estrogen receptor signaling pathways via TGF-beta was shown to be mediated by SMAD3, which indicates a role of *SMAD3* in breast cancer progression (50). Furthermore, we identified a DMP located on chromosome 21 in the BTB and CNC homology1 (*BACH1*) gene. *BACH1* encodes a transcription factor that is upregulated in tumours from triple-negative breast cancer (TNBCs) patients (51). *BACH1* has been previously reported as a regulator of metastasis in TNBCs and its gene signature was shown to predict poor outcomes in breast cancer (52). The promoter of *TNIP1* was differentially methylated and transcriptionally dysregulated in the T21-BC group. The tumor necrosis factor α–induced protein 3–interacting protein 1 (*TNIP1)* is part of the NF-κB and RAR signaling pathways (53, 54). *TNIP1* was one of the stromal genes exhibiting expression changes when comparing adenomas vs cancer-associated stroma (55). Two DMRs were identified in *KRBOX4* and *TOM1* in the tiling analysis located in promoter flanking regions. *KRBOX4* is located on the X chromosome and no studies so far have provided any link to breast cancer. *TOM1* is required for autophagosome maturation and endosomal trafficking (56). *TOM1* additionally represses Toll-like receptor signalling and plays a role in immune receptor recycling (57, 58). Mutations in *TOM1* have been recently shown to be associated with early-onset autoimmunity and combined immunodeficiency (59). In addition, it is important to mention that we did not observe any DNA methylation changes at the region level in GTPases of the immunity-associated proteins (GIMAPs) despite their recently identified tumour suppressive role against breast cancer in DS (33). Therefore, it seems that upregulation of *GIMAPs* in T21 women is not associated with changes in DNA methylation.

DS patients are known to exhibit strong epigenetic age acceleration and for this reason we tested T21-BC patients age acceleration in comparison to the T21-BCF group. Epigenetic age acceleration have been previously shown to occur in several diseases including cancer, and can be used as a potential biomarker for early disease detection (26). Furthermore, a longitudinal study reported epigenetic age acceleration measured on blood DNA to be associated with a higher risk of developing breast cancer (60). The PhenoAge clock was also shown to measure increased epigenetic age acceleration in breast tissue of from breast cancer patients. However, our analysis revealed no difference in epigenetic age acceleration between T21-BC and T21-BCF using various clocks. This might be related to the drastic increase in epigenetic age acceleration in DS, which masks the effects of breast cancer on DNA methylation age.

The limitations of our study are the small sample size, however this is related to the uniqueness of the condition since breast cancer is almost non-existent in T21 patients. Furthermore, we have only looked at blood DNA in this study, which might not reflect similar epigenetic changes to target tissues involved in disease pathogenesis. Therefore, future studies should include additional tissues to determine whether the observed epigenetic changes related to breast cancer in DS are systemic or only restricted to one tissue.

In conclusion, this is the first study to investigate the DNA methylation profile in Down syndrome women suffering from breast cancer. The identified differentially methylated genes/regions could help us better understand factors that protect against breast cancer, which can provide new avenues for potential therapeutic targets or preventive approaches.

## Data availability statement

The raw data supporting the conclusions of this article will be made available by the authors, without undue reservation.

## Ethics statement

The studies involving human participants were reviewed and approved by Institutional Review Board Statement. This research was conducted according to the guidelines of the Declaration of Helsinki. It was approved by the Institute Jérôme Lejeune Committee on Clinical Investigation and the Institutional Review Board (IRB) of the Qatar Biomedical Research Institute (reference number: QBRI-IRB 2021-07-098). Written informed consent was obtained from the parents or guardians prior to the participants being included in the research study. The patients/participants provided their written informed consent to participate in this study.

## Author contributions

AM and NH designed the study; YB and SA analyzed the data, performed experiments, and prepared figures; SD, MV, LM, and AM collected the samples and clinical data; NH and AA wrote the manuscript, NH, AM, and AA reviewed and edited the manuscript. All authors contributed to the article and approved the submitted version.
