# Supplementary figures and images for: DNA methylation profiling in Trisomy 21 females with and without breast cancer
Source: Front Oncol. 2023 Jul 19;13:1203483. doi: 10.3389/fonc.2023.1203483 (PMC10395079; doi:10.3389/fonc.2023.1203483)

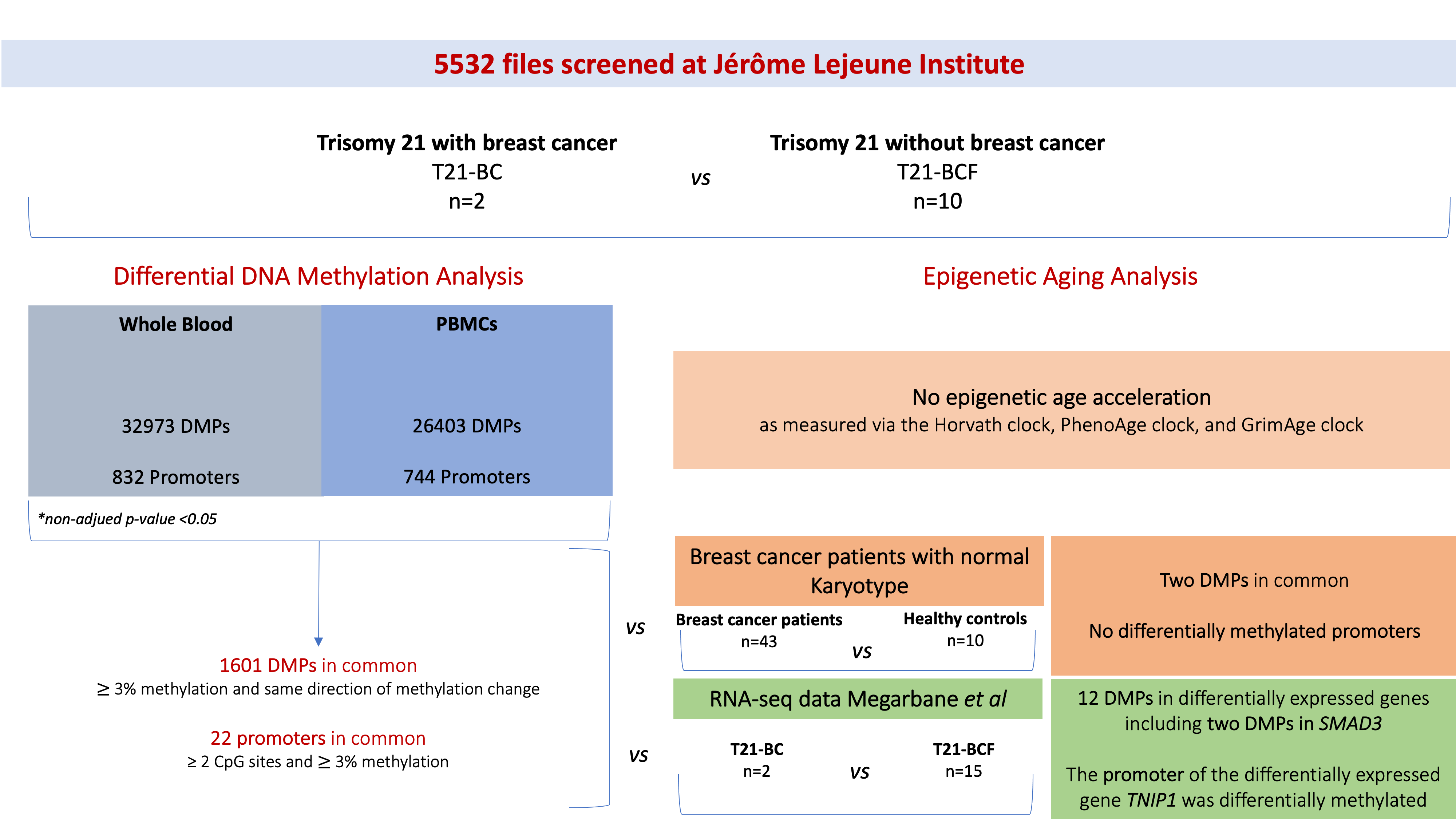

Supplement: Supplementary file 1 [file Image_1.tiff]
